# Effect of Protein Thermal Denaturation on the Texture Profile Evolution of Beijing Roast Duck

**DOI:** 10.3390/foods11050664

**Published:** 2022-02-24

**Authors:** Yanxia Liu, Zhenyu Wang, Dequan Zhang, Teng Pan, Huan Liu, Qingwu Shen, Teng Hui

**Affiliations:** 1 Key Laboratory of Agro-Products Processing, Ministry of Agriculture and Rural Affairs, Institute of Food Science and Technology, Chinese Academy of Agricultural Sciences, Beijing 100193, China; liuyanxia@henau.edu.cn (Y.L.); wangzhenyu@caas.cn (Z.W.); noahpan99@163.com (T.P.); liuhuan02@caas.cn (H.L.); huiteng@caas.cn (T.H.); 2 International Joint Laboratory of Meat Processing and Safety in Henan Province, College of Food Science and Technology, Henan Agricultural University, Zhengzhou 450002, China; 3 Henan Key Laboratory of Meat Processing and Quality Safety Control, College of Food Science and Technology, Henan Agricultural University, Zhengzhou 450002, China; 4 College of Food Science and Technology, Hunan Agricultural University, Changsha 410128, China; yaoyao3153@aliyun.com

**Keywords:** Beijing roast duck, protein thermal denaturation, texture profile, roasting

## Abstract

To investigate the mechanism of the texture formed by protein thermal denaturation, the profile and formation of texture and thermal denaturation of protein were evaluated using texture profile analysis (TPA) and transmission electron microscopy (TEM) combined with differential scanning calorimeter (DSC). Results indicated that the surface temperature of Beijing roast duck increased from 23.9 to 174.4 °C, while the center temperature rose from 20.6 to 99.3 °C during roasting. Shear force decreased significantly during the first 20 min, and the texture profile largely changed at 20 and 40 min. Firstly, Band I was broken and twisted, Band A was overstruck, and Z-line was diffused and finally disappeared, resulting in a blurred myofibril structure. The sarcomere considerably contracted within 30 min. Secondly, the main myofibrillar proteins were denatured at 20 and 40 min, respectively. The formation of hydrophobic interactions and the reduction of ionic bonds were observed. Thirdly, roasting induced protein thermal denaturation, which was correlated with interprotein forces, texture profile, and the shear force. Muscle fibers were damaged and shrunken, accompanied by the formation of hydrophobic interactions and the reduction of ionic bonds. The turning points were at 20 and 40 min, and the main proteins were denatured, leading to the formation of tenderness of Beijing roast duck.

## 1. Introduction

Beijing roast duck is a kind of traditional Chinese food, which is very popular among consumers [1]. Traditionally, the roasting process of Beijing roast duck is empirically controlled by the operators, and how to maintain the traditional texture after industrial production is a great challenge for the industrialization of Beijing roast duck. Tenderness is one of the important indicators of roast duck and is a general summary of the structural characteristics of proteins. High correlations have been found between heat-, glycolysis-, energy metabolism-, muscle fiber structure-, oxidation-, and degradation-related protein and meat tenderness or tenderization [2,3].

When meat is exposed to heat, proteins will denature and coagulate, thereby reducing the space of myofibrils, and finally forming the texture of the product [3]. Roasting time and temperature affect protein aggregation, muscle fiber contraction, and sarcomere length, and then eventually affect meat tenderness. Different shear forces and textures were formed under different roasting temperatures, and meat was less tender after roasting in general. A good correlation between tenderness and the heat treatment process was found in some reports [4,5].

Hydrolysates produced by protein degradation are abundant in meat [6], and some of these tenderness-related proteins differ greatly between tender and tough samples [7]. The muscle fiber was found to be broken, and the connective tissue was intact after roasting [8]. The temperature and time of roasting had a great influence on the denaturation and contraction of the myofibril [4]. The hydrophobicity and aggregation of proteins in meat increased after heat treatment [9,10]. Actin, myosin heavy chain, and sarcoplasmic protein are aggregated after roasting [11]. Whereas myosin light chain and tropomyosin are heat-stable [3,4,9] and the polymerization of proteins is increased, and heating induces the cleavage of proteins near aspartic acid residues [12]. No noticeable degradation of myosin has been observed at 80 °C, and actin did not degrade during heating [4]. However, the thermal denaturation of meat proteins and its influence on the texture properties of processed products during roasting, such as Beijing roast duck, are still not well-understood. Beijing ducks were processed according to traditional techniques, such as air inflation, blanching, drying, coloring, etc., and finally hung in the oven for roasting by the experienced roast duck chef. The traditional hanging ovens (Gua-lu) differ greatly from other ovens in that they have an arched mouth but no door. The duck is hung on an iron hook in the oven, and fruitwood is used to roast the duck by fire.

Therefore, it is essential to study the change of temperature, protein, and muscle fiber structure during roasting, and thus the texture change rule of traditional Beijing roast duck. This will be conducive to the application of new manufacturing technologies and the development of new roasting equipment, and to further realize the industrialization and intelligent transformation of roast duck.

## 2. Materials and Methods

### 2.1. Sample Preparation

The raw duck was supplied by Beijing Jinxing Ducks Co., Ltd. (Jiugong Town, Daxing District, Beijing, China). The same batch of Beijing four-series stuffed ducks, 42 days old, were used as experimental materials, and the weight of each duck was 2000 ± 300 g. After slaughter, the ducks were cooled and then transported to the restaurant for processing. The ducks were processed according to traditional techniques at Dongxinglou restaurant in Beijing (located in Dongzhimen, Chaoyang District). Air inflation was a necessary step in which air was pumped into the duck between the skin and subcutaneously, making the duck look appealing. Then, boiling water (100 °C) was poured onto the surface of the duck 3 times, and the time of this procedure was controlled to 3~5 s. After blanching, the duck was dried for 2~4 h in a room at 2~4 °C. To make the body color acceptable, caramel and maltose were used before freezing. The mass ratio of caramel and maltose to water was 1:8. Afterward, the ducks were frozen at −5 °C for 2–3 days to make the skin of the duck thicker and more delicious. Before roasting, the ducks were thawed at 2~4 °C and heated to room temperature. Finally, the ducks were hung in the traditional oven (Gua-lu) for roasting by the experienced roast duck chef.

**Figure 1:**
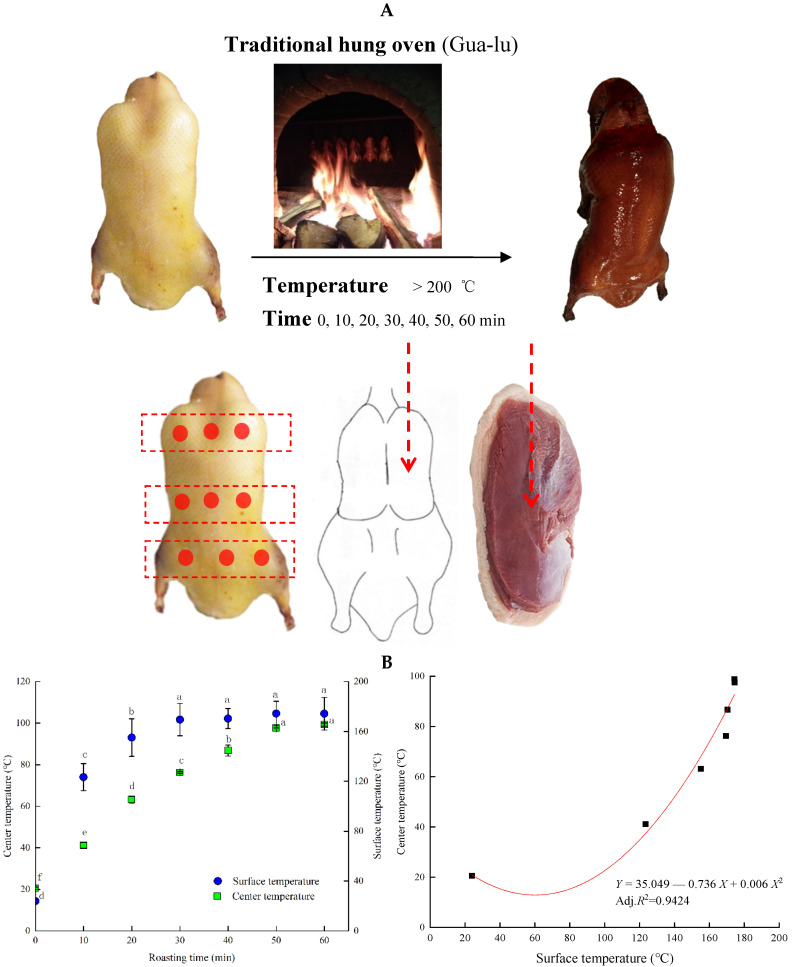
Changes in the surface temperature and center temperature of the Beijing roast duck during roasting. The thermograph probes were inserted into the middle of the duck breast for determining the center temperature. The surface temperature was measured with an infrared detector. Means (±SE) of surface temperature and center temperatures for the Beijing roast duck roasted for different times (0–60 min) are shown (**A**). The different letters indicate that the center temperature and surface temperature showed significant differences (*p <* 0.05). The fitting equation of the center temperature and surface temperature was obtained by using Origin software and the actual monitoring points are represented by black dots (**B**).

The top and bottom temperatures of the oven were between 202 and 203 °C, and the center temperature was 224 °C during roasting. According to experience, the roast duck chef determined that the roasting time of the experimental duck was 60 min. Six ducks were removed from the oven at 0, 10, 20, 30, 40, 50, and 60 min, respectively, and the duck breast muscles were separated and measured immediately. Beijing duck is roasted with fruitwood as fuel in a closed oven (Men-lu) or a hanging oven (Gua-lu). Gradually, the Gua-lu procedure dominated the preparation practice of Beijing roast duck and was further refined in the imperial kitchen of the Qing Dynasty (1644–1911). The arched mouth of the oven characterizes the Gua-lu, and no door exists. The duck is hung on an iron hook in the oven, and fruitwood (pear or jujube wood is the best) is used to roast the duck by fire [13]. Figure 1 shows how Beijing roast duck is processed according to traditional technology (Gua-lu) and how the experiment was designed. The surface temperature of the duck was measured 3 times with an infrared detector every 10 min, and the specific monitoring points are shown in Figure 1. The center temperature was continuously observed by a multichannel temperature inspector, and the probe position is shown in Figure 1. To obtain the temperature rise law of the duck breast, Origin software was used to carry out model fitting.

### 2.2. Texture Profile Evolution

Shear force and texture profile analysis as well as microstructure analysis are usually used to evaluate the tenderness of meat products, and shear force and texture profile analysis represent macro-changes, while microstructure analysis indicates the formation of micro-texture profile evolution. The samples in this experiment were directly cooled after roasting for shear force, texture profile, and microstructure measurement, without freezing.

#### 2.2.1. Shear Force

**Figure 2:**
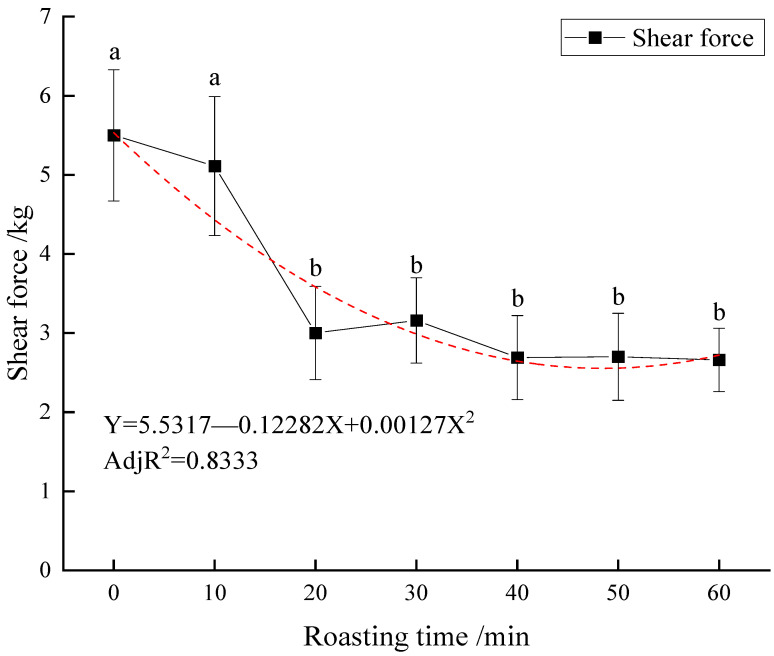
Changes of shear force of Beijing roast duck during roasting. Shear force (kg) for Beijing roast duck breast roasted for different times (0–60 min). Data are presented as Mean ± SE. Shear force values lacking a common letter differ (*p <* 0.05). The fitting equation of shear force with roasting time was obtained using Origin software.

The duck breast meat was cut into 3 × 1 × 1 cm sections along the direction of the muscle fiber, and the shear force was measured using a TA-XT2i texture analyzer (Stable Micro Systems Ltd., Godalming, UK), referring to the description of Wyrwisz et al. with slight modifications [14]. The test parameters were as follows: the cutter probe was adopted with a 25 kg arm, the pretest speed was 2 mm/s, the feed speed was 1 mm/s, and the feed distance was 33 mm. The results were analyzed using Texture Expert Exceed 2.64 software, which was provided by the instrument manufacturer (Stable Micro Systems Ltd., Godalming, UK). A total of ten replicates were measured, and the average value was obtained. The shear force (kg) for Beijing roast duck breast roasted for different times is shown in Figure 2.

#### 2.2.2. Texture Profile Analysis

Samples were cut into 1 × 1 × 1 cm sections along the direction of the muscle fiber, and a TA-XT2i texture analyzer (Stable Micro Systems Ltd., Godalming, UK) was used to measure the texture profile. The number of replicates was ten, and the data were averaged. Measurements were conducted according to a published report [15], with some modifications. The diameter of the probe was 50 mm, the compression ratio was 70%, the induction force was 5.0 g, the interval time was 5.0 s, and the trigger type was automatic. The speeds of the pretest, in-test, and posttest were 2.0, 1.5, and 1.0 mm/s, respectively. Hardness, adhesiveness, springiness, cohesiveness, gumminess, chewiness, and resilience were calculated using Texture Expert Exceed 2.64 software, which was supplied by the instrument manufacturer (Stable Micro Systems Ltd., Godalming, UK). The texture profile analysis of Beijing roast duck during Gua-lu roasting is shown in Table 1.

### 2.3. Microstructure Analysis

The microstructure was based on the method of Zhang et al., with slight modifications [16], using transmission electron microscopy (TEM). The muscles were cut into strips (10 × 1 × 1 mm) according to the direction of myofibrils, and 2.5% glutaraldehyde fixative solution (pH 7.2) was immediately added to fix for one week. Samples were rinsed 3 times (10 min each time) with phosphate buffered solution (PBS, pH 7.4) and then fixed in 1% osmium tetroxide for 2–3 h. Afterward, the samples were rinsed 3 times (10 min each time) again and then dehydrated with 30%, 50%, 60%, 70%, 80%, 90%, and 100% ethanol solution for 7–15 min at each concentration, respectively, with constant agitation. The solvent was then switched to acetone, and the samples were mixed with increasing concentrations of epoxy (33%, 50%, 66%, 2 h intervals). After polymerization (12 h at 37 °C, 12 h at 40 °C, 12 h at 45 °C, 12 h at 50 °C, and 48 h at 60 °C) and cooling at room temperature for more than 2 days, a UC6 ultrathin slicing machine was used for ultrathin slicing. The slices were stained with uranium acetate and lead citrate (0.4% lead citrate, 0.3% lead nitrate, and 0.3% uranium acetate). Pictures were collected using a transmission electron microscope (Hitachi LTD, Tokyo, Japan). More than ten sarcomere lengths were measured by Image-pro-plus (Media Cybernetics Inc., Rockville, MD, USA) for each fiber. The microstructure of Beijing roast duck during roasting is shown in Figure 2.

### 2.4. Protein Thermal Stability

Protein thermal stability can be reflected by protein thermal denaturation, which was measured by a differential scanning calorimeter (DSC) (Perkin Elmer Co., Ltd., Waltham, MA, USA), according to Zhao et al.’s [17] method with slight modifications. The isolated samples were placed in aluminum pans after accurately weighing and hermetically sealing. A 15 mg sample was placed into an aluminum box for sealing. Then, it was put into the DSC instrument for determination. Air was used as a reference gas, and nitrogen was used as a protective gas. An empty pan was used as a reference. The samples were balanced at 20 °C for 2 min and then heated at a rate of 5 °C·min^−^^1^ to 100 °C. Thermograms were recorded for estimating peak temperature, peak area, and enthalpy (ΔH). Calculations were performed to determine the mean and standard deviations of four replicate samples. Protein thermal denaturation of Beijing duck during roasting is shown in Table 2.

### 2.5. Intermolecular Forces of Actomyosin

Samples were prepared according to Glorieux’s method with some modifications [18]. One hundred grams of ground meat was placed in a 500 mL beaker. Then, 300 mL of 0.05 M KCl potassium phosphate buffer (0.02 M K_2_HPO_4_, 0.02 M KH_2_PO_4_, pH 7.0) was added, and a high-speed homogenizer was applied at a speed of 10,000 r·min^−1^. The homogenate was allowed to stand for 1 min, followed by 30 s for homogenization. The mixed slurry was then transferred to a 500 mL centrifuge tube and centrifuged at 10,000× *g* for 5 min at 4 °C. The precipitate was collected and centrifuged again. Next, 300 mL of 0.6 M KCl phosphoric acid buffer (0.02 M K_2_HPO_4_, 0.02 M KH_2_PO_4_, pH 7.0) was added to the precipitate, which was then homogenized at 10,000 r·min^−1^ for 30 s, dispersed for 1 min, centrifuged (10,000× *g* × 5 min, 4 °C), and homogenized at 10,000 r·min^−1^ for 30 min. Then, 600 mL of potassium phosphate buffer (0.02 M K_2_HPO_4_, 0.02 M KH_2_PO_4_, pH 7.0) was used to dilute the filtrate. The liquid was stirred evenly by a magnetic stirrer for 30 min and then centrifuged (10,000× *g* × 10 min, 4 °C). The precipitate was resuspended in 100 mL of 0.05 M KCl phosphate buffer (0.02 M K_2_HPO_4_, 0.02 M KH_2_PO_4_, pH 7.0) and centrifuged (10,000× *g* × 10 min, 4 °C). The precipitate was actomyosin. Then, samples were stored at 4 °C and analyzed immediately.

**Figure 3:**
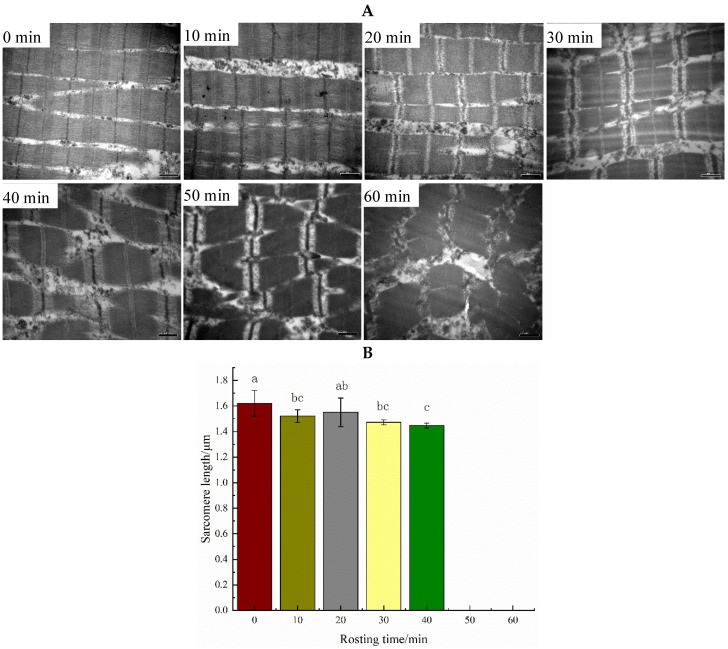
Changes of microstructure and sarcomere of Beijing roast duck during roasting, as shown by (**A**) transmission electron microscopy (TEM) photographs, together with the sarcomere length (**B**). Beijing roast duck breasts were roasted for different times (0–60 min). The microstructure was obtained by transmission electron microscopy of 30,000 times. Sarcomere length could not be measured after roasting for 50 min or longer. Bars lacking a common letter differ (*p <* 0.05).

Ten milliliters of 40 mg/mL actomyosin solutions were sealed in a 10 mL centrifuge tube and heated to the inner temperature of duck breast meat, such as 20.6, 63.2, 86.8, and 99.3 °C, which corresponded to roasting times of 0, 20, 40, and 60 min, respectively. According to the methods used by Gómez-Guillén, Liu, and Ni [19,20,21], the solutions were treated with chemicals to cleave certain kinds of bonds. Each treatment was measured four times. Variations of intermolecular forces of actomyosin are shown in Figure 3.

### 2.6. Statistical Analysis

SPSS, Origin, and Image-pro-plus were used to process the experimental data. The analysis of variance and the relationship were generated using one-way ANOVA and Bivariate correlation of SPSS 22.0 (IBM Corporation Inc., New York, NY, USA), respectively. Figure 1, Figure 2 and Figure 3 were drawn and performed by Origin 8.6.0 (OriginLab Corporation, Rockville, MD, USA). The sarcomere length was spatially calibrated and measured by Image-pro-plus 6.0 (Media Cybernetics Inc., Rockville, MD, USA). The roasting time (0, 10, 20, 30, 40, 50, and 60 min) was considered a fixed effect, and the breast muscles from the roasted Beijing ducks were considered a random effect. The results are expressed as the mean ± standard deviation.

## 3. Results

### 3.1. The Center and Surface Temperature of Duck Breast Meat

As shown in Figure 1, during roasting at 220–230 °C, the surface temperature of Beijing roast duck increased from 23.9 to 174.4 °C, while the center temperature rose from 20.6 to 99.3 °C within 60 min. The surface temperature of the ducks increased fast in the first 10 min, with 99.5 °C elevation, then changed slightly after 30 min. Whereas the center temperature increased by 42.6 °C rapidly within 20 min, then kept stable after 50 min. The center temperature took a longer time to stabilize than the surface temperature. The center temperature is closely related to the degree of protein denaturation and the integrity of muscle structure. The ending center temperature of roasting has a great influence on the tenderness, color, and flavor of the Beijing roast duck, and is the key control point of standardized production. In order to obtain the relationship between the surface temperature and the center temperature, the prediction equation of Beijing roast duck was established by Origin software. The increasing tendency of the center temperature was fitted by a nonlinear fitting (*p <* 0.05, Figure 1). The fitting equation of the center temperature (*Y*) with the surface temperature (*X*) was *Y* = 35.049 − 0.736 *X* + 0.006 *X*² (Adj. *R*^2^ = 0.9424). The equation fitting was significant.

### 3.2. Changes in Shear Force and Texture Profile Analysis

Consumer acceptance of meat is strongly influenced by the eating quality, and tenderness is thought to be the most important characteristic of eating quality. Shear force has been widely used to estimate the tenderness of raw and cooked meat. The shear force decreased significantly from 5.50 (53.9 N) to 3.00 kg (29.4 N) after 20 min of roasting (*p <* 0.05), then changed slightly from 20 to 60 min (*p >* 0.05, Figure 2). The center temperature rose to 63.2 °C at 20 min, indicating that the first 20 min was the critical time for the formation of shear force. In order to obtain the relationship between the shear force and the roasting time, the prediction equation of the Beijing roast duck was established. The decreasing tendency of shear force was fitted by a polynomial fitting method (*p <* 0.05, Figure 2). The fitting equation of shear force (*Y*) with roasting time (*X*) was *Y* = 5.53170 − 0.12282 *X* + 0.00127 *X*^2^ (Adj *R*^2^ = 0.8333).

As shown in Table 1, all texture indexes of the duck breast muscle changed significantly (*p <* 0.05) with different variation trends during roasting. The hardness, chewiness, and gumminess of roast duck meat first increased and then decreased. Adhesiveness, springiness, and cohesiveness increased while resilience decreased with roasting time (*p <* 0.05). The hardness increased from 6221.15 to 9394.92 g in the first 20 min (*p <* 0.05). Similarly, for gumminess and chewiness, they also increased to the maximum at 20 min, and the maximums were 4249.31 and 1957.54 N, respectively. In addition, adhesiveness decreased from 15.06 to 6.43 N·s (*p <* 0.05), reaching the maximum (16.40 N·s) at 20 min and the minimum (6.43 N·s) at 60 min, but the values did not change after 30 min (*p >* 0.05). Similarly, springiness values increased from 0.38 to 0.45 mm in the first 20 min (*p <* 0.05), and to the maximum of 0.52 mm at 40 min, and remained stable after 20 min (*p >* 0.05). Cohesiveness values increased from 0.37 to the maximum of 0.48 at 40 min (*p <* 0.05) and did not change after 20 min (*p >* 0.05). In contrast, resilience values decreased from 0.24 at the beginning to the minimum of 0.15 at 60 min and did not change after 40 min (*p <* 0.05).

### 3.3. Microstructure Changes

The myofibrils of fresh duck breast muscle were well-organized. The M-line, Z-line, Band A, Band I, and H zones were visible, and no significant change was observed in the first 10 min of roasting, and during the next 20–40 min, Band I shrinkage, M-line and Band A overstriking, and Z-line diffusion were observed (Figure 3A). Moreover, the Z-line disappeared, Band I broke, and the M-line was visible after roasting for 50 min. In the end, Band I broke and twisted, and the myofibril structure became blurred.

After roasting, the sarcomere of breast meat contracted, and the sarcomere length decreased significantly from 1.62 to 1.47 microns within 30 min (*p* *<* 0.05). Additionally, the sarcomere length could not be measured after continuous roasting for 50 min (Figure 3B).

### 3.4. Changes in Intermolecular Forces of Myofibrillar Proteins

**Figure 4:**
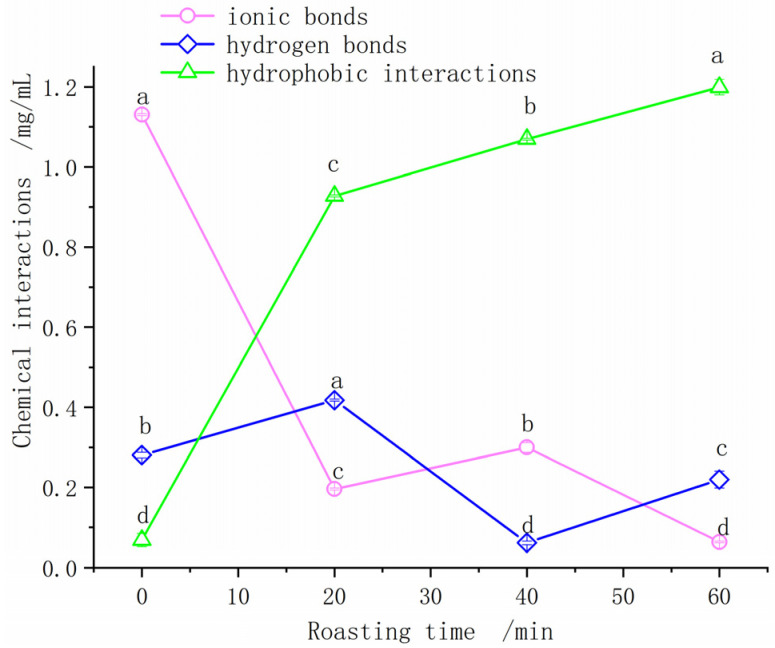
Variations of intermolecular forces of actomyosin. Solutions were heated to the inner temperature of duck breast meat, such as 20.6, 63.2, 86.8, and 99.3 °C, corresponding to the roasting times of 0, 20, 40, and 60 min, respectively. Of the same parameter, values lacking a common letter differ (*p <* 0.05).

Molecular interactions, including ionic bonds, hydrogen bonds, and hydrophobic interactions of actomyosin in solution, were analyzed to study their contribution to the formation and maintenance of the gel structure (Figure 4). The ionic bond of actomyosin in duck breast muscle decreased with the largest variation after roasting for 20 min (*p <* 0.05), and then rose slightly at 40 min and decreased after 40 min, which indicated that actomyosin aggregated. The hydrogen bond first showed a rising and then reducing trend and reached a maximum at 20 min and a minimum at 40 min (*p <* 0.05). It was suggested that the higher temperature would make the ionic bond and hydrogen bond unstable. In addition, the hydrophobic interaction increased gradually as well, and the rising rate was large at 20–40 min (*p <* 0.05).

### 3.5. Analysis of Protein Thermal Stability

Differential scanning calorimetry (DSC) was adopted to reflect the unfolding condition and protein denaturation degree of muscle protein. Peak I represents myosin head degeneration caused by heat flux changes (approximately 55 °C), peak II represents myosin changes in the tail and muscle plasma protein denaturation (approximately 60~64 °C), and peak III represents actin denaturation (approximately 78 °C) [21,22]. The results of the DSC determination of duck breast meat are shown in Table 2. It was observed that the thermal denaturation of proteins occurred step-by-step during roasting. The degree of protein denaturation gradually intensified with increasing temperature. There were 3 peaks in the duck meat samples after roasting for 10 min, and peak I and peak II disappeared after roasting for 10 and 20 min, respectively, which indicated the denaturation of the myosin head and the myosin tail, respectively. Accordingly, the main proteins contained in the meat were entirely denatured and data were not shown after 40 min.

## 4. Discussion

### 4.1. Effects of Temperature on Protein Denaturation and Interprotein Forces

**Figure 5:**
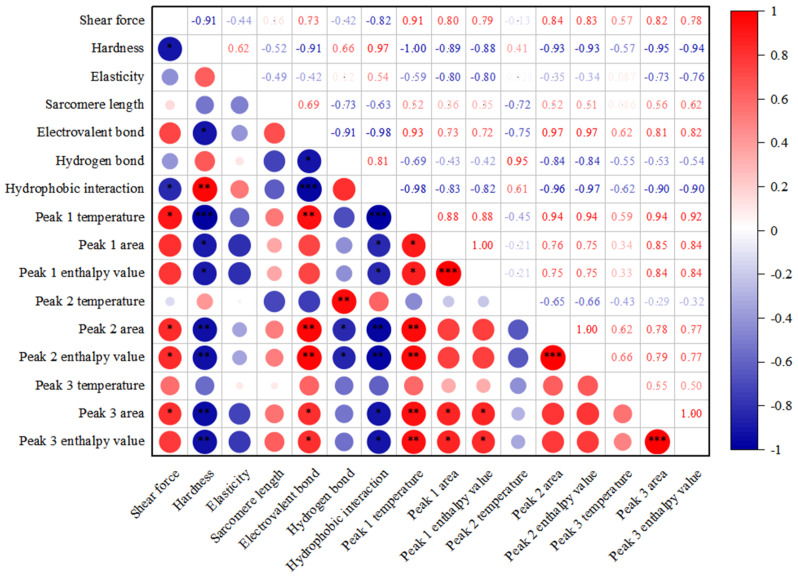
Correlation analysis between protein thermal denaturation and texture profile evolution of Beijing duck during roasting. Correlation coefficient between two indicators is shown. *, **, and *** indicate significant difference in correlation coefficients *p <* 0.05, *p <* 0.01, and *p <* 0.001, respectively. The size of the circle is determined by the value of the correlation coefficient. If the correlation coefficient is high, the circle is large, and vice versa. Blue represents the negative correlation between indicators, while red represents the positive correlation. The higher the correlation coefficient is, the darker the color will be, otherwise, it will be lighter.

In this study, the denaturation of myosin in roast duck occurred between 10 and 20 min after roasting, and the center temperature was 63.2 °C at 20 min, indicating that myosin in meat was denatured at or under this temperature. However, the thermal denaturation of actin happened between 30 and 40 min, at which time the center temperature was 86.8 °C. After 40 min, most of the main proteins were completely denatured. The ionic bond of actomyosin decreased, and the decline rate was large at 0–20 min, but it increased slightly at 40 min and even started to decline again after 40 min (*p <* 0.05). The hydrogen bond reached the maximum at 20 min and the minimum at 40 min (*p <* 0.05). The results were consistent with the protein thermal degeneration, which showed that myosin head thermal degeneration occurred at 20 min and that the main myofibrillar proteins denatured completely at 40 min. The hydrophobic, aromatic amino acid side chain was exposed to the protein surface, changed the protein conformation, and eventually led to the formation of hydrophobic interactions, which was consistent with previous studies [11,23,24,25,26]. In addition, the ionic bond was positively correlated with the temperature of peak I as well as the area and enthalpy (ΔH) of peaks II and III, the hydrogen bond was positively correlated with the temperature, area, and enthalpy (ΔH) of peak II, and the hydrophobic interaction was negatively correlated with the temperature, area, and enthalpy (ΔH) of peak I as well as the area and enthalpy (ΔH) of peaks II and III (*p <* 0.05, Figure 5). The results showed that intermolecular forces were related to major myofibrillar protein thermal denaturation during roasting (*p <* 0.05), which was consistent with a previous study [20]. When meat is heated, protein is thermal denatured and functional groups such as hydrophobic groups are unfolded and exposed. Meanwhile, hydrogen bonds and ionic bonds are formed, which could result in the aggregation of proteins and the emergence of a three-dimensional network [10,27,28,29,30,31,32,33]. However, the turning points of duck breast meat were different with various final cooked temperatures [34].

Therefore, although the traditional roasting is inconsistent with the industrial procedure, the relationship between protein denaturation and the center temperature is in line with the normal law, so the key point to achieve industrialization is to control the center temperature, and find the relationship between the industrial heat treatment temperature and the center temperature.

### 4.2. Microstructural Changes Caused by Protein Denaturation and Interprotein Forces

Muscle fibers are the building blocks of muscle. The overall structure of myofibrils shrinks after heating, and the shrinkage might be along the direction of vertical or parallel myofibrils, but the degree is different. Myofibril lateral shrinkage occurs mainly at 45–60 °C, while longitudinal contraction occurs at 60–90 °C [35,36,37,38]. Transverse shrinkage of the fiber in low-temperature long-term (LTLT)-treated pork *Longissimus dorsi* increased in order between 53 and 59 °C [35]. In this study, after roasting for 20–40 min, Band I shrinkage, M-line and Band A overstriking, and Z-line diffusion were observed, and the Z-line disappeared, but Band I broke, and the M-line was visible after roasting for 50 min. With the increasing roasting time, the sarcomere of breast meat contracted (*p <* 0.05, Figure 3). Sarcomere length decreased continuously from 1.62 to 1.47 microns within 30 min (*p <* 0.05), and the overall structure tended to be close. Palka et al. [36] found that the myofibril structure was destroyed by increasing the roasting time. After roasting for 50 min, the structure of the myofibrils of the Beijing duck breast muscle was damaged, and sarcomere length could not be measured (Figure 3). However, heat distribution damaged the surface of muscle fibers and caused some fiber shrinkage, which was still visible at 100 °C [37]. It was suggested that this difference could be attributed to different raw materials and heat resistance. Roasting causes more structural damage than other cooking methods, and protein thermal denaturation causes some fiber shrinkage [8]. Roasting time has a significant effect on muscle fiber shrinkage [39]. In addition, the sarcomere length was positively correlated with the ionic bond, and negatively related to the hydrophobic bond (*p <* 0.05, Figure 5), which was consistent with previous reports [3,40].

The three-dimensional structure of myofibrils such as myosin and actin is maintained by hydrophobic interaction, hydrogen bond, electrostatic interaction, van der Waals force, and disulfide bond. Heat could change the interprotein forces, and then lead to protein denaturation [4], furthermore causing structural changes of muscle fibers [17]. With the increase of temperature, the hydrophobic interaction becomes stronger, while the hydrogen bond, van der Waals force, and electrostatic interaction become weaker, which makes most proteins unfold. The double-helix structure of denatured myosin expands, and then forms a special extension structure [41]. At the same time, the hydrophobic group is exposed outward. Further myosin aggregates and shortens the sarcomere [42]. Although the heating method and raw materials are different, the change of interprotein interaction force and the protein denaturation that led to the shrinkage degree of muscle fiber are different, but they all follow similar rules.

### 4.3. Mechanism of Texture Formation during Roasting

Protein thermal denaturation and unfolding and exposure of functional groups, such as hydrophobic groups, occurs when muscle proteins are heated. At the same time, hydrogen bonds and ionic bonds form, which could result in the aggregation of proteins and the emergence of a three-dimensional network; finally, texture profile evolution is formed [10,27,28,29,30,31,42]. The relationships of the key indicators in this study were shown in Figure 5. The results demonstrated that the shear force was only highly negatively correlated with hydrophobic interactions, which was inconsistent with the negative correlation between shear force and sarcomere length [34]. In comparison, there was a strong positive correlation between shear force and ionic bond and temperature, area, and enthalpy (ΔH) of peak III (*p <* 0.05). Hardness was positively correlated with hydrogen bonds and the temperature of peak I (*p <* 0.05). Springiness was negatively correlated with sarcomere length and temperature, area, and enthalpy (ΔH) of peak I (*p <* 0.05). Sarcomere length was negatively correlated with hydrophobic interactions (*p <* 0.05), but positively correlated with ionic bonds (*p <* 0.05), which disagreed with the high correlation between the shrinkage of sarcomere and the tenderness of duck meat [34]. Ionic bonds were negatively correlated with hydrophobic interactions but positively correlated with main protein denaturation (*p* *<* 0.05). Hydrogen bonds were highly correlated with the temperature, area, and enthalpy (ΔH) of peak II (*p <* 0.05). Hydrophobic interactions were negatively correlated with the temperature, area, and enthalpy (ΔH) of peak I as well as the area and enthalpy (ΔH) of peaks II and III (*p <* 0.05). In general, protein thermal denaturation was negatively correlated with hardness, springiness, hydrophobic interactions, and hydrogen bonds (*p <* 0.05), and positively correlated with shear force and ionic bonds. Therefore, there might be some interaction between protein thermal denaturation and texture profile evolution during roasting.

Roasting affects the texture due to protein thermal denaturation. Roasting time has a significant effect on the tenderness of beef muscles [39]. Mitra et al. [3] pointed out that a longer cooking time could result in the variation of texture. Barbanti et al. [5] reported that a short cooking time results in the best meat tenderness. Shear force decreased with the increasing roasting time (*p <* 0.05, Figure 2), which disagrees with the results found by Li et al. [34] and Wattanachant et al. [37]. Li et al. [34] reported that shear force was found to increase in two separate phases, from an internal temperature of 40–50 °C and again from 60 to 95 °C, with a decrease from 50 to 60 °C. The shear value of chicken muscles significantly increased from 50 to 80 °C, but did not change from 80 to 100 °C [37]. The difference might be attributed to the variations in raw materials, protein thermal denaturation, and processing methods. We once tried to evaluate the tenderness of roast duck meat according to the shear force. The shear force decreased significantly from 5.50 kg (53.9 N, namely hard) to 2.66 kg (26.1 N, namely very tender) during roasting. However, no unified standard for studying tenderness was established, which agreed well with a previous study by Pathare et al. [32]. Some texture profile indexes, such as hardness, chewiness, and gumminess, first increased and then decreased, and adhesiveness, springiness, and cohesiveness increased, while resilience decreased (*p <* 0.05, Table 2), which was similar to previous results [5,39,43]. There was no significant correlation between protein thermal denaturation and texture profile (*p <* 0.05, Figure 5), which indicated that actin denaturation had a stronger effect on shear force changes, and myosin denaturation had a stronger impact on texture profile analysis. The results showed that actin denaturation had a more substantial effect on texture profile changes than myosin denaturation, which was consistent with published studies [44,45].

Heat treatment, such as roasting, could lead to changes in the intermolecular forces of proteins [11,24,25,26], and the protein denatures as heating temperature increases [19], further causing some fiber shrinkage [8,32,33,39,40] and the formation of gel in proteins at the same time [20,21,26], finally resulting in the formation of texture [3,45].

## 5. Conclusions

Results of this study showed that protein thermal denaturation was responsible for the texture profile evolution and the delicacy of the Beijing roast duck. The equation of surface temperature and center temperature could realize the real-time prediction of roast duck center temperature, and furthermore guide the equipment parameter setting during the industrialization and develop of new roasting equipment, finally realizing the industrialization and intelligent transformation of Beijing roast duck. Actin denaturation had a more significant effect on texture profile changes than myosin denaturation, and intermolecular forces were related to main myofibrillar protein thermal denaturation during roasting. Myosin thermal degeneration was conducted at 20 min, and the main myofibrillar proteins denatured completely at 40 min; therefore, 20 and 40 min were the turning points, and the temperatures corresponding to these two time points were also the key control temperatures. In order to form the traditional texture of Beijing roast duck, the center temperature should reach nearly 100 °C within 60 min. However, the difference between traditional Beijing roast duck and industrialized products at the same roasting temperature needs to be further verified. Further studies of the relationship between myofibrin and other proteins such as elastin and sarcoplasmin, as well as between proteins and fat, are still needed.

## Figures and Tables

**Table 1:** Change of texture profile analysis of Beijing roast duck during Gua-lu roasting.

Roasting Time/min	Hardness/g	Adhesiveness/N·s	Springiness/mm	Cohesiveness	Gumminess	Chewiness/N	Resilience
0	6221.15 ± 1581.25 ^b^	−15.06 ± 3.47 ^ab^	0.38 ± 0.06 ^c^	0.37 ± 0.07 ^b^	2372.63 ± 806.90 ^b^	966.06 ± 305.66 ^b^	0.24 ± 0.05 ^ab^
10	7719.40 ± 1606.31 ^ab^	−14.10 ± 3.79 ^abc^	0.38 ± 0.08 ^bc^	0.37 ± 0.06 ^b^	3150.43 ± 1032.03 ^ab^	1303.33 ± 348.11 ^ab^	0.24 ± 0.06 ^ab^
20	9394.92 ± 1628.89 ^a^	−16.40 ± 4.29 ^ab^	0.45 ± 0.07 ^a^	0.44 ± 0.05 ^ab^	4249.31 ± 837.18 ^a^	1957.54 ± 412.57 ^a^	0.21 ± 0.04 ^b^
30	6819.49 ± 1092.67 ^b^	−8.62 ± 2.11 ^c^	0.51 ± 0.05 ^a^	0.47 ± 0.06 ^a^	3197.49 ± 373.18 ^ab^	1626.57 ± 217.55 ^a^	0.20 ± 0.03 ^b^
40	6725.24 ± 1081.84 ^b^	−9.20 ± 3.03 ^bc^	0.52 ± 0.06 ^a^	0.48 ± 0.07 ^a^	3221.04 ± 513.96 ^ab^	1677.39 ± 322.01 ^ab^	0.19 ± 0.04 ^bc^
50	7887.75 ± 1240.04 ^ab^	−10.04 ± 3.55 ^abc^	0.51 ± 0.08 ^a^	0.44 ± 0.04 ^ab^	3468.84 ± 612.06 ^ab^	1844.42 ± 320.75 ^ab^	0.16 ± 0.02 ^c^
60	7085.69 ± 968.19 ^ab^	−6.43 ± 3.91 ^c^	0.47 ± 0.08 ^ab^	0.45 ± 0.06 ^ab^	3253.19 ± 538.99 ^ab^	1563.02 ± 332.22 ^ab^	0.15 ± 0.03 ^c^

Note: Mean ± standard deviation. Within the same column, means lacking a common letter differ (*p <* 0.05).

**Table 2:** Protein thermal denaturation of Beijing duck during roasting.

Roasting Time/min	Peak Ⅰ			Peak Ⅱ			Peak Ⅲ		
	Temperature/°C	Area/mJ	ΔH/J/g	Temperature/°C	Area/mJ	ΔH/J/g	Temperature/°C	Area/mJ	ΔH/J/g
0	55.10 ± 0.48 ^a^	1.05 ± 0.62 ^a^	0.07 ± 0.04 ^a^	62.02 ± 1.10 ^b^	3.06 ± 0.69 ^b^	0.20 ± 0.05 ^b^	78.12 ± 0.14 ^ab^	4.63 ± 0.95 ^a^	0.31 ± 0.06 ^a^
10	36.99 ± 27.74 ^b^	0.74 ± 0.07 ^a^	0.05 ± 0.04 ^a^	61.25 ± 1.12 ^b^	5.78 ± 4.54 ^a^	0.40 ± 0.33 ^a^	78.29 ± 0.19 ^a^	4.45 ± 1.44 ^a^	0.30 ± 0.10 ^a^
20	UD	UD	UD	63.71 ± 1.13 ^a^	1.23± 0.39 ^bc^	0.08 ± 0.03 ^bc^	78.24 ± 0.36 ^ab^	3.90 ± 0.71 ^a^	0.25 ± 0.05 ^a^
30	UD	UD	UD	UD	UD	UD	78.02 ± 0.12 ^b^	1.85 ± 1.98 ^b^	0.12 ± 0.13 ^b^

Note: The measurement results of 40, 50, and 60 min showed no peak. Mean ± standard deviation. Within the same column, means lacking a common letter differ (*p <* 0.05). UD = undetectable.

## Data Availability

The data presented in this study are available upon request from the corresponding author.
